# Graphene Quantum Dots with High Yield and High Quality Synthesized from Low Cost Precursor of Aphanitic Graphite

**DOI:** 10.3390/nano10020375

**Published:** 2020-02-21

**Authors:** Shuling Shen, Junjie Wang, Zhujun Wu, Zheng Du, Zhihong Tang, Junhe Yang

**Affiliations:** School of Materials Science and Engineering, University of Shanghai for Science and Technology, Shanghai 200093, Chinaduzhengduyilin@163.com (Z.D.); zhtang@usst.edu.cn (Z.T.)

**Keywords:** graphene quantum dots, aphanitic graphite, low cost precursor, high yield, high quality

## Abstract

It is difficult to keep the balance of high quality and high yield for graphene quantum dots (GQDs). Because the quality is uncontrollable during cutting large 2D nanosheets to small 0D nanodots by top-down methods and the yield is low for GQDs with high quality obtained from bottom-up strategy. Here, aphanitic graphite (AG), a low-cost graphite contains a large amount of small graphite nanocrystals with size of about 10 nm is used as the precursor of graphene oxide quantum dots (GO-QDs) for the first time. GO-QDs with high yield and high quality were successfully obtained directly by liquid phase exfoliating AG without high strength cutting. The yield of these GO-QDs can reach up to 40 wt. %, much higher than that obtained from flake graphite (FG) precursor (less than 10 wt. %). The size of GO-QDs can be controlled in 2–10 nm. The average thickness of GO-QDs is about 3 nm, less than 3 layer of graphene sheet. Graphene quantum dots (GQDs) with different surface properties can be easily obtained by simple hydrothermal treatment of GO-QDs, which can be used as highly efficient fluorescent probe. Developing AG as precursor for GQDs offers a way to produce GQDs in a low-cost, highly effective and scalable manner.

## 1. Introduction

Graphene, with unique thermal, mechanical, and electronic properties, as well as its excellent chemical stability, has attracted increasingly attention in recent years [1,2,3]. These superior properties of graphene have advanced applications in energy, catalysis, and biomaterial fields [4,5,6,7,8,9]. However, large graphene nanosheet has zero bandgap which limits its electronic and opto-electronic application [10,11]. According to quantum confinement and edge effect, the bandgap of graphene can be designed by varying its size. GQDs with zero-dimensional structure, higher photo stability, lower toxicity, and easily changeable functional groups have attracted considerable attention for applying in sensors, bioimaging, and solar cell [12,13,14,15,16,17,18].

Over the past few years, GQDs have been widely studied and various synthesis methods have been developed [19,20,21]. These methods can be divided into two types: bottom-up strategy and top-down strategy. As for bottom-up approach, solution-based chemical routes were demonstrated as useful methods to synthesize large polycyclic aromatic hydrocarbons from dendritic arene precursors [22,23,24]. Li group employed 2′,4′,6′-triakyl phenyl groups as the precursor to form the edge of graphene moieties [25]. Through this approach, the size and colloidal stability could be controlled. But bottom-up approaches usually contain many complicated steps and the yield of GQDs is low. Furthermore, during the synthesis process, large amount of organic solvents is usually used. The purification of obtained GQDs is difficult and the purified GQDs cannot be dispersed in water easily, which limit their applications. Comparing with bottom-up strategy, top-down approach is a relatively simple process. The crucial point of this approach is how to cut large 2D nanosheets to small 0D nanodots. Many researchers are committed to developing simple and efficient methods for cutting large sheets into nanodots. Various synthesis routes have been developed, such as oxidation cutting [26], hydrothermal (solvothermal) [27,28], electrochemistry [29,30,31], photo-Fenton reaction [32], and oxygen plasma etching [33]. Various precursors were adopted, such as graphene oxide (GO) [34], C60 [35], carbon nano-onions [36], carbon fibers [37], coal [38,39], graphite nanoparticle [40], and rice husk [41]. However, there are also many problems that limit the practical application of GQDs, such as complex pretreatments, high energy-consumption, special instruments or high precursor costs, and so on. It is still a challenge to synthesize GQDs or GO-QDs at a large scale with uniform size in a high yield and low-cost way.

Different from coal, AG is a kind of graphite ore that is composed of carbonaceous material by thermal decomposition of deep metamorphic products (such as from coal deterioration). Its conductivity, thermal conductivity, lubricity, and oxidation resistance are lower than full crystalline graphite. Therefore, it has not been taken seriously and its price is much lower than flake graphite. AG with particle size of ~5 μm has been used to synthesize small size graphene sheets [42,43]. But it is ignored that AG contains a large amount of small graphite nanocrystals with size of about 10 nm. Here, reserve thinking of how to cut 2D graphene sheets into 0D nanodots efficiently, we report for the first time the large-scale synthesis of GO-QDs with uniform size by simply exfoliating AG precursor through a chemical exfoliation process without high-strength cutting. The yield of GO-QDs can reach up to 40%, much higher than that by using FG as precursor (less than 10%). GQDs with different surface properties can be easily obtained by simply hydrothermal treating of GO-QDs in the presence of different functional molecules. These modified GQDs can be used as highly efficient fluorescent probe.

## 2. Materials and Methods

### 2.1. Synthesis of GO-QDs by Exfoliating of AG

The exfoliating of AG (Chenzhou Botai Graphite Co., Ltd., Hunan, China) was carried out by a conventional intercalation process, which was generally used for preparing large GO sheets. In a typical procedure, 5 g of AG, 2.5 g of NaNO_3_, and 115 mL of concentrated H_2_SO_4_ were ultrasonically mixed in a beaker at room temperature for 30 min. Then the beaker was removed into an ice bath under mild agitation and 15 g of KMnO_4_ was added slowly at a temperature under 10 °C. Then the mixture was heated to 35 °C and kept for 40 min. After adding 250 mL of deionized (DI) water, the solution was kept at 98 °C for another 45 min. Then the mixture was diluted to 700 mL and 30 mL of H_2_O_2_ (30%) was added. After that, the solution was centrifuged and washed by DI water for several times until the pH value of the system reached to about 7. Then the as-prepared sample was first centrifuged at 3000 rpm for 15 min, dividing into supernatant and precipitates. The precipitates mainly composed of un-exfoliated AG were removed. The obtained GO-QDs were dispersed in water for further study. To make a comparison, FG (CP, Aladdin Reagent Database Inc., Shanghai, China) was exfoliated via the same procedure. For investigating the exfoliating mechanism of AG, the exfoliating process was stopped at intercalation and oxidation steps, respectively. The corresponding intermediate products were isolated, characterized, and stored under appropriate conditions. The products obtained after intercalation by H_2_SO_4_ are defined as AG-I and FG-I, respectively, and the products obtained after oxidation by KMnO_4_ are defined as AG-O and FG-O, respectively.

### 2.2. Preparation of GQDs with Different Functional Groups

For obtaining GQDs, 1 mg of the prepared GO-QDs was added into 10 mL of deionized water, and the mixture was placed into a sonic bath for 30 min to obtain a homogeneous solution. The solution was sealed in a Teflon-line autoclave at 90 °C–200 °C for 12 h. After cooling to room temperature, GQDs were obtained. For obtaining GQDs modified with functional groups, different functional molecules can be added during the hydrothermal process. For example, modified GQDs of GQDs-NH_3_·H_2_O, GQDs-NaBH_4_, and GQDs-H_3_BO_3_ were simply prepared by adding ammonia, NaBH_4_, and H_3_BO_3_ as modifier during hydrothermal treatment, respectively.

### 2.3. Characterization

The crystalline phases of the samples were identified by using X-ray powder diffraction (XRD) techniques (Bruker D8 Advance diffractometer, Karlsruhe, Germany) operating with Cu Kα radiation (λ = 0.15418 nm) at a scan rate (2θ) of 3 o·min^−1^. The scan range is from 10° to 70°. The morphologies of the samples were characterized by using a field emission scanning electron microscope (SEM, Hitachi S4800, Tokyo, Japan), and a Tecnai G2 F30 S-Twin transmission electron microscope (TEM, FEI company, Hillsboro, OR, USA). The morphology and thickness of GO-QDs were obtained by an atomic force microscope (AFM, Cypher ES, Asylum Research, Oxford Instrument, Oxford, UK). Samples used for AFM characterization were prepared by dipping the piranha-cleaned silicon wafer substrate into their ethanolic suspensions. Raman spectra were recorded using a Raman microscopy (Horiba, LabRAM HR Evolution, Villeneuve-d’Ascq, France). Thermogravimetric analyses (TG) of samples were conducted using a thermal analyzer (NETZSCH, Selb, Germany). Samples were analyzed in the temperature range of 25–790 °C under a flow of nitrogen. X-ray photoelectron spectroscopy (XPS) was carried out on a PHI-5000 Versa Probe instrument (ULVAC-PHI, Chigasaki, Japan) with Al Kα X-ray source. The Fourier transform infrared spectra (FI-IR) of the samples were obtained with a PerkinElmer spectrum 100 (PerkinElmer, Waltham, MA, USA), in which KBr was used as diluents. PL lifetimes and quantum yield were investigated by LSP920 (Edinburgh Instruments, Corston, UK) using Xenon lamps (450 W).

### 2.4. Detection of Metal Ions in Water

Cu^2+^ solution was prepared in advance with CuCl_2_, and then mixed with the above-mentioned GQDs-NH_3_·H_2_O in a certain proportion. Finally, a series of solution were obtained with a concentration of Cu^2+^ in the range of 0–100 μM. The resulting solution was shaken well to ensure the complete reaction, and then its fluorescence emission is measured at the optimal excitation wavelength of 323 nm to detect the change of fluorescence intensity because of Cu^2+^. For detecting other metal ions, CuCl_2_ was replaced by FeCl_3_, MnCl_2_, AgNO_3_, KCl, CuCl_2_, CaCl_2_, BaCl_2_, MgCl_2_, CdCl_2_, and ZnCl_2_, respectively.

## 3. Results and Discussion

### 3.1. Synthesis and Characterization of GO-QDs from AG

**Figure 1:**
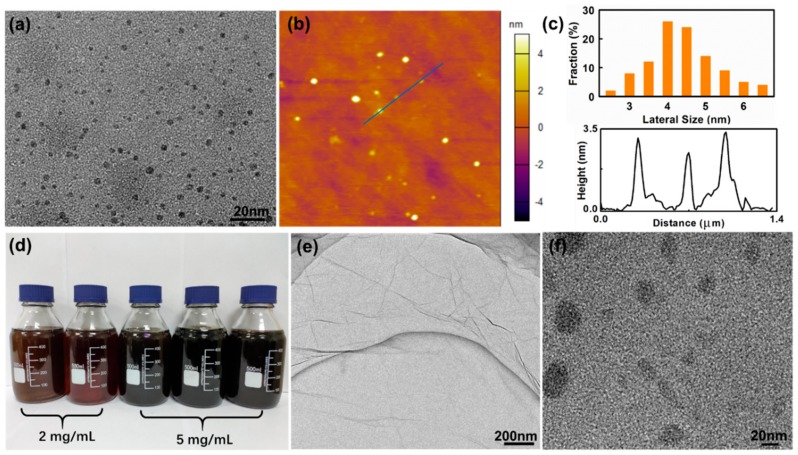
(**a**) Transmission electron microscope (TEM) and atomic force microscope (AFM). (**b**) Images of graphene oxide quantum dots (GO-QDs) synthesized from aphanitic graphite (AG). (**c**) Lateral size and height distribution. (**d**) Mass produced GO-QDs aqueous solution. (**e**) TEM image of GO synthesized from flake graphite (FG). (**f**) GO nanosheets synthesized from FG. (Separated at 15,000 rpm for 30 min).

The exfoliating of AG was carried out by a modified Hummers’ method, which is the most commonly used for the preparation of graphene oxide (GO) sheets [44]. TEM and AFM images in Figure 1a,b indicate that product synthesized from AG is composed of quantum dots with narrow size distribution. The average size of these GO-QDs is about 4.5 nm as calculated by counting 100 nanoparticles (Figure 1c). GO-QDs have an average thickness of ~3 nm, which corresponds to only 2–3 layers of graphene sheets (Figure 1c). The yield of these GO-QDs is about 40 wt. % of raw AG. So, a large amount of GO-QDs can be obtained with AG precursor, and the concentration of GO-QDs aqueous solution can reach up to 5 mg·mL^−1^ (Figure 1d). For comparison, FG were also adopted as precursors for preparing GO-QDs under the same conditions. Figure 1e shows that at low resolution the product synthesized from FG looks like a crumpled membrane with large size, which should be GO sheets. Small size quantum dots cannot be observed on this large crumpled membrane. If this product is further separated under 15,000 rpm for 30 min, large GO nanosheets are removed and small size GO nanosheets can be observed (Figure 1f). The size of these small GO nanosheets is mostly larger than 30 nm and not uniform, as well as the yield of these small GO sheets is less than 10 wt. % of raw FG. Above results demonstrate that GO-QDs with high yield and high quality can be synthesized by using low cost AG.

### 3.2. Synthesis Mechanism of GO-QDs from AG

**Figure 2:**
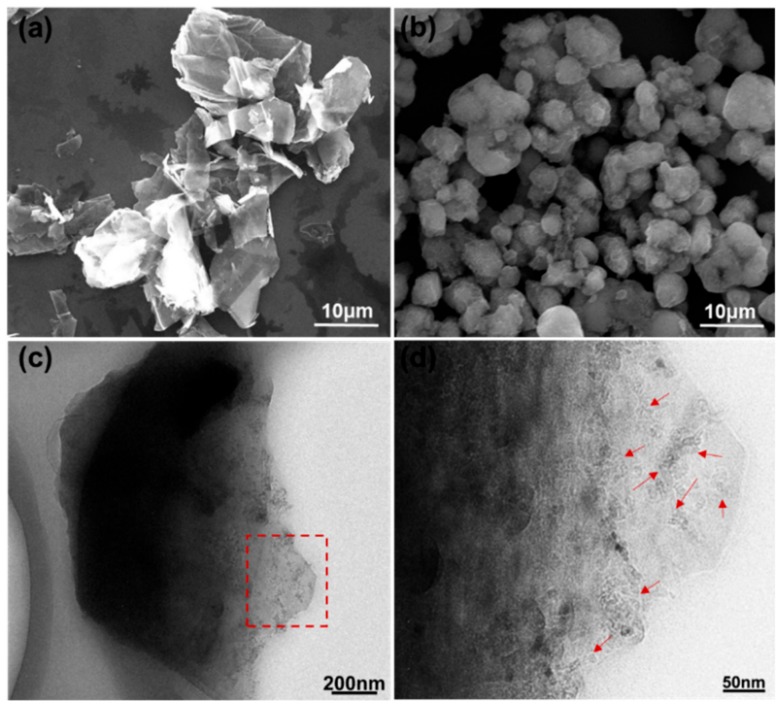
(**a**) SEM image of FG, (**b**–**d**) SEM and TEM images of aphanitic graphite (AG). The red arrows in Figure 2d point the small graphite nanocrystals.

To understand the synthesis mechanism of GO-QDs from AG, the difference between AG and FG were compared first. The morphology difference between AG and FG can be obviously observed from their SEM images. Figure 2a indicates that FG has a large layered structure. The average length of these layers is about 35 μm. Completely different from FG, AG has a particle structure (Figure 2b). The average diameter of AG particles is about 5 μm (Figure 2c). It is noteworthy that each AG particle contains a large amount of small graphite nanocrystals with size of less than 10 nm as pointed in Figure 2d. These graphite nanocrystals are not fully developed graphite crystals during the formation period. If these small graphite nanocrystals are exfoliated, GQDs will be obtained directly without needing high-intensity or repeated cutting.

To further investigate the structural difference, XRD and Raman spectroscopy of AG and FG were characterized. Figure S1a shows XRD patterns of AG and FG. It can be observed that there are two strong peaks at 26.5° and 54.6° in the XRD pattern of FG, which is characteristic peaks of (002) and (004) of graphite materials [45]. In the XRD pattern of AG, the intensity of these peaks is quite weaker than that of FG, which suggests the low c-axis order and poor crystallinity of AG. The positions of characteristic peaks of AG are the same as that of FG and no other impurities are detected in AG, suggesting the graphite nature of AG but not amorphous coal. The Raman spectrum of FG in Figure S1b reveals their defect-free graphite nature due to the absence of D bond signal. In AG, D band (1341 cm^−1^) is strong and I_D_/I_G_ ratio is 0.48 attributing to smaller average size of sp2 domains in AG. The smaller the sp2 domain, the higher the edge concentration in AG is and the stronger the D band. These results are in accordance with the TEM and SEM results.

**Figure 3:**
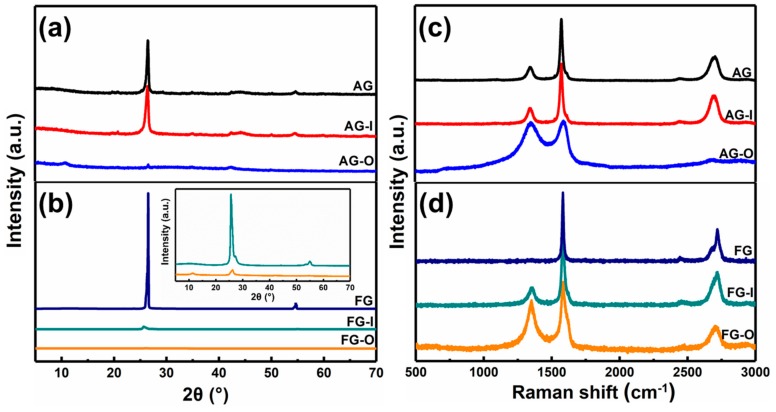
(**a**) XRD patterns and (**c**) Raman spectra of AG, AG-I, AG-O. (**b**) XRD patterns and (**d**) Raman spectra of FG, FG-I, and FG-O.

Tour and co-workers showed that the formation of GO from bulk graphite by modified Hummers’ method is a diffusive-controlled process [46]. Smaller-size flakes and lower crystalline graphite are oxidized significantly faster and more fully than the large and high crystalline flakes at same usage of oxidant. For investigating the mechanism in depth, the chemical and structural changes, which occurred during exfoliating of AG and FG, were monitored by XRD, Raman, FTIR, and TG analyses. The products obtained after intercalation by H_2_SO_4_ are defined as AG-I and FG-I, respectively, and the products obtained after oxidation by KMnO_4_ are defined as AG-O and FG-O, respectively. Figure 3 show XRD patterns and Raman spectra of AG, AG-I, AG-O, FG, FG-I, and FG-O. The corresponding data are listed in Table 1. 

**Figure 4:**
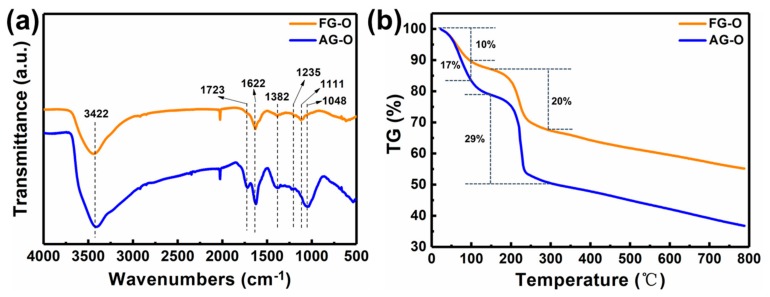
(**a**) FTIR spectra and (**b**) TG curves of FG-O and AG-O.

**Figure 5:**
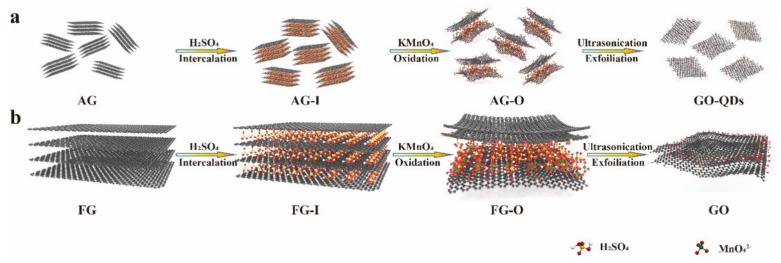
Schematic of the exfoliating of (**a**) AG and (**b**) FG with the same amount of intercalator and oxidizer.

It can be observed that intercalation process has less effect on the structure of AG. However, for FG, after intercalation of H_2_SO_4_, the intensity of characteristic peak of (002) is dramatically decreased and I_D_/I_G_ ratio increases to 0.71, indicating an increase of degree of disorders. The less effect of intercalation on AG is related to the more disorders of AG than FG. After oxidation, the characteristic peaks at 26.5° in AG and FG almost disappear and new peaks appear at 10.71° and 11.34°, respectively (Figure 3a,b). From Figure 3c,d and Table 1 it can be found that after oxidation I_D_/I_G_ ratios of AG-O and FG-O all significantly increase compared with that of the corresponding precursors, indicating the destruction of conjugated sp2 networks. The I_D_/I_G_ ratio of AG-O is much higher than that of FG-O, suggesting more oxygen-containing functional groups in AG-O than FG-O. The higher-degree oxidation of AG is also confirmed by FTIR and TG analyses in Figure 4. FG-O exhibits many vibration peaks of O−H stretch and bending at 3422 cm^−1^ and 1382 cm^−1^, respectively, attributed to hydroxyl and phenolic groups, C=C stretch of unoxidized sp2 carbon domain at 1622 cm^−1^, C–O stretch at 1235 cm^−1^ ascribed to phenols, ethers, and epoxy groups, and C–O stretch at 1048 and 1111 cm^−1^ attributed to hydroxyl groups (Figure 4a). All these mentioned peaks can be observed in AG-O, and they are stronger than those in FG-O. Additionally, C=O stretch at 1723 cm^−1^ attributed to carboxyl and carbonyl groups is detected in AG-O. TG analysis indicates that both FG-O and AG-O show two significant weight losses (∼10 and ∼17%) near 100 °C and (∼20 and ∼29%) in the range of 150–300 °C, owing to the evaporation of the trapped water molecules and thermal decomposition of oxygen-containing functional groups in the samples, respectively (Figure 4b). All above results demonstrate that AG has a higher degree oxidation. The oxidation is complete and homogeneous due to shorter diffusion path for oxidant between AG layers, which is difficult to achieve for FG at the same condition because of the diffusion resistance between large layers. A large amount of GO-QDs can be obtained by exfoliation of fully oxidized AG-O without further high-strength cutting (Figure 5).

### 3.3. Modification of GO-QDs and Their Applications

**Figure 6:**
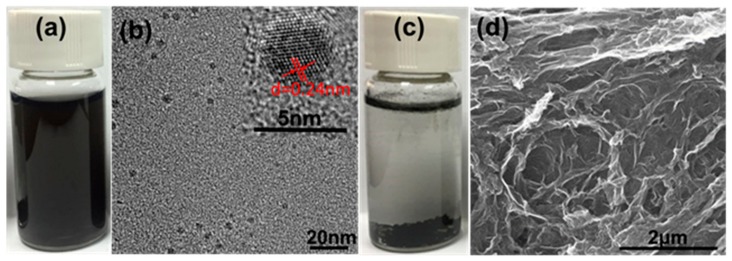
(**a**) Picture and (**b**) TEM and HRTEM (inset) images of GQDs by hydrothermal treatment of GO-QDs. (**c**) Picture and (**d**) SEM image of product after hydrothermal treatment of GO.

The obtained large amount of GO-QDs from AG can be used as precursor for GQDs with different properties. If GO-QDs are treated in pure water by hydrothermal method, brown GO-QDs turn into black GQDs. These GQDs can keep in good dispersion in water even after standing for 6 months (Figure 6a). TEM image in Figure 6b further confirms the monodispersing of GQDs. The concentration of the obtained GQDs dispersion can reach to 1 mg/ml. HRTEM image of GQDs was measured as shown in Figure 6b inset. The lattice distance of 0.24 nm is observed, suggesting the crystalline nature of GQDs. Controlled experiment was carried out by treating the product synthesized from FG at the same condition. The picture in Figure 6c indicates that after hydrothermal treatment the black agglomerate deposits at the bottom. During hydrothermal treatment, the large GO sheets (Figure 1e) with rich -OH and -COOH groups in the plane are very easy to connect with each other and form a 3D structure by the interaction of hydrogen bond [47] (Figure 6d). 

**Figure 7:**
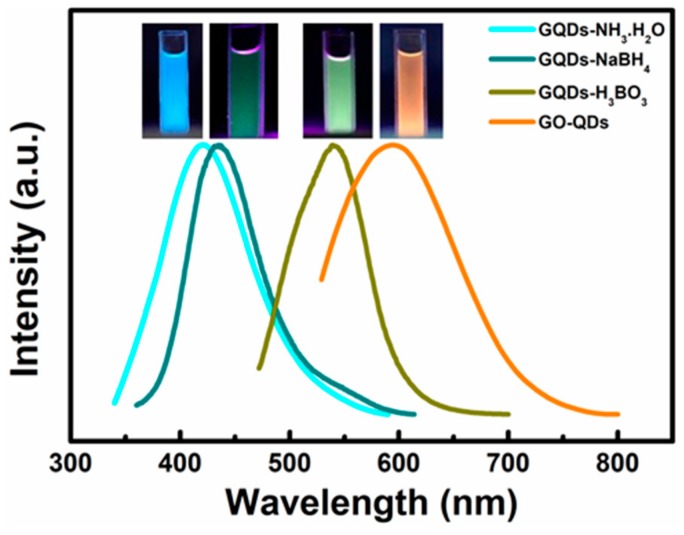
PL spectra of GO-QDs, GQDs-NH_3_·H_2_O, GQDs-NaBH_4_ and GQDs-H_3_BO_3_ excited at 323 nm. The insets are their corresponding digital photographs (λex = 365 nm).

**Figure 8:**
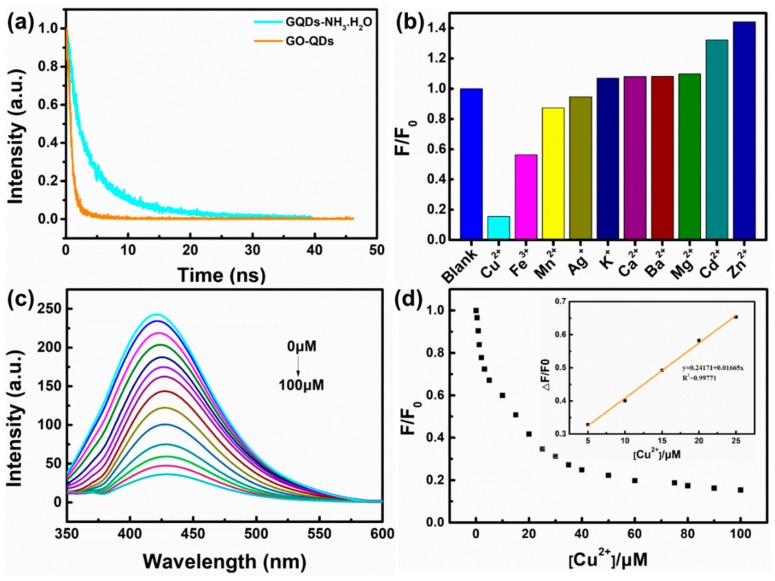
(**a**) Time resolved PL decay curves of GO-QDs and GQDs-NH_3_·H_2_O. (**b**) The effect of different metal ions on the PL intensity of GQDs-NH_3_·H_2_O, (**c**) PL quenching of GQDs-NH_3_·H_2_O with different concentration of Cu^2+^, and (**d**) the relationship of F/F_0_ and Cu^2+^ concentration.

XPS test further confirm the reduction of GO-QDs during hydrothermal treatment. Comparing with GO-QDs (Figure S2a), the binding energy peaks of GQDs do not shift, but their intensities drastically change. The intensities of oxygen-containing functional groups such as -C-O, -C=O, and -O-C=O significantly decrease. The peak intensity of C-C increases (Figure S2b). These results suggest the reduction of GO-QDs during hydrothermal treatment. GO-QDs has an orange-emission at 595 nm (Figure 7). After the removal of oxygen-containing functional groups, GQDs almost have no fluorescence emission. While a series of GQDs with different fluorescence properties can be easily synthesized from GO-QDs by adding modifiers during hydrothermal treatment. For example, three kinds of soluble fluorescent GQDs could be obtained by adding ammonia, NaBH_4_, and H_3_BO_3_ as modifier during hydrothermal treatment, respectively. These fluorescent GQDs are defined as GQDs-NH_3_·H_2_O, GQDs-NaBH_4_ and GQDs-H_3_BO_3_, respectively. These modified GQDs exhibit fluorescence emission at 425 nm, 435 nm, and 539 nm, respectively (Figure 7). Therefore, GQDs with different fluorescence properties for specific application requirements can be synthesized by AG in a large scale and its quality and quantity can be guaranteed by the special precursor. For example, the lifetime of GO-QDs is 1.07 ns and that of GQDs-NH_3_·H_2_O is 5.98 ns. Meanwhile, the quantum yield of GQDs-NH_3_·H_2_O (9.7%) is about twenty-five times as much as that of GO-QDS (0.4%) (Figure 8a and Table 2). After modification, the size of GQDs-NH_3_·H_2_O is almost the same as that of GO-QDs (Figure S3a). FTIR spectrum of GQDs-NH_3_·H_2_O demonstrates obvious N–H stretching vibration peaks in the range of 3300–3600 cm^−1^, N-H bending peak at 1655 cm^−1^ and C–H stretching vibration peak at 1412 cm^−1^ (Figure S3b). These results indicate the successful modification of GO-QDs by NH_3_·H_2_O. GQDs-NH_3_·H_2_O with higher quantum yield can be used as fluorescence probe for detecting metal ions in water. The results indicate that GQDs-NH_3_·H_2_O has a high selectivity for copper ions (Cu^2+^) (Figure 8b). Figure 8c displays the PL intensity spectra of GQDs-NH_3_·H_2_O with different concentration of Cu^2+^ at 323 nm excitation. It can be clearly observed that the fluorescence quenching of GQDs-NH_3_·H_2_O is naturally increasing with the increasing of Cu^2+^ concentration. The modified plot in Figure 8d shows the relationship of F/F_0_ and Cu^2+^ concentration. There is a distinct linear relationship between quenched PL intensity (ΔF/F_0_) and Cu^2+^ concentration in the range of 5 μM to 25 μM (Inset in Figure 8d). The limitation of detection can reach up to 0.4 μM, which is enough to satisfy the demand in detection for Cu^2+^ in wastewater.

## 4. Conclusions

In conclusion, AG was used for the synthesis of GQDs for the first time. Comparing with FG, the merits of AG as precursor for GQDs is as follows: (i) AG is low cost, (ii) the synthesis process does not need high-strength cutting step, (iii) the size of obtained GQDs is uniform, high yielding (~40%), and of good quality, (iv) can be easily modified according to the request of applications. It is believed that these GQDs synthesized by AG as precursor can be a candidate for graphene-based tunable luminescent material in many potential fields.

## Figures and Tables

**Table 1:** Comparation of XRD and Raman data of AG and FG during exfoliating.

Measurements		Pristine	After Intercalation	After Oxidation
2θ(°)	AG	26.5	26.4	10.7
	FG	26.5	25.7	11.3
I_D_/I_G_	AG	0.48	0.60	2.65
	FG	0	0.71	1.31

**Table 2:** Exponential fitting results for time resolved PL decay curves of GO-QDs and GQDs-NH_3_·H_2_O.

Samples	τ_1_(ns)	A_1_(%)	τ_2_(ns)	A_2_(%)	Lifetime(ns)
GO-QDs	0.6056	75.95	2.5346	24.05	1.07 ns
GQDs-NH_3_·H_2_O	2.1349	36.59	8.1989	63.41	5.98 ns

## Supplementary Materials

The following are available online at https://www.mdpi.com/2079-4991/10/2/375/s1. Figure S1: (**a**) XRD patterns and (**b**) Raman spectra of AG and FG. Figure S2: XPS patterns of GO-QDs (**a**) and GQDs (**b**). Figure S3: (**a**) TEM image of GQDs-NH_3_·H_2_O.and (**b**) FTIR spectra of GQDs-NH_3_·H_2_O and GO-QDs.
