# Supplementary material for: Graphene Quantum Dots with High Yield and High Quality Synthesized from Low Cost Precursor of Aphanitic Graphite
Source: Nanomaterials (Basel). 2020 Feb 21;10(2):375. doi: 10.3390/nano10020375 (PMC7075322; doi:10.3390/nano10020375)
Supplement: Supplementary file 1 [file nanomaterials-10-00375-s001.pdf]

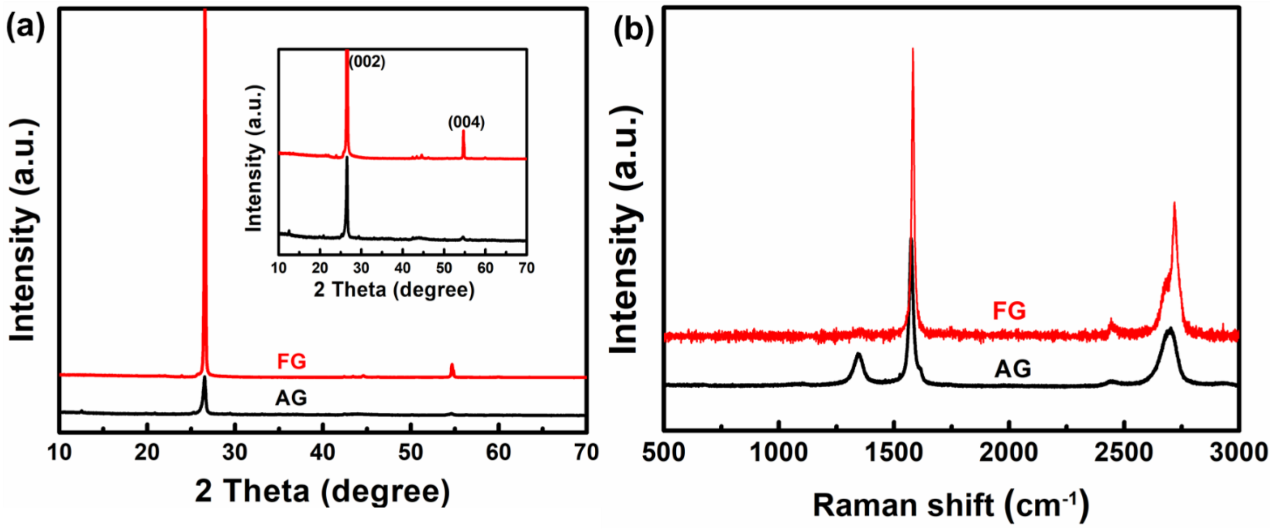

Figure S1. (**a**) XRD patterns and (**b**) Raman spectra of AG and FG.

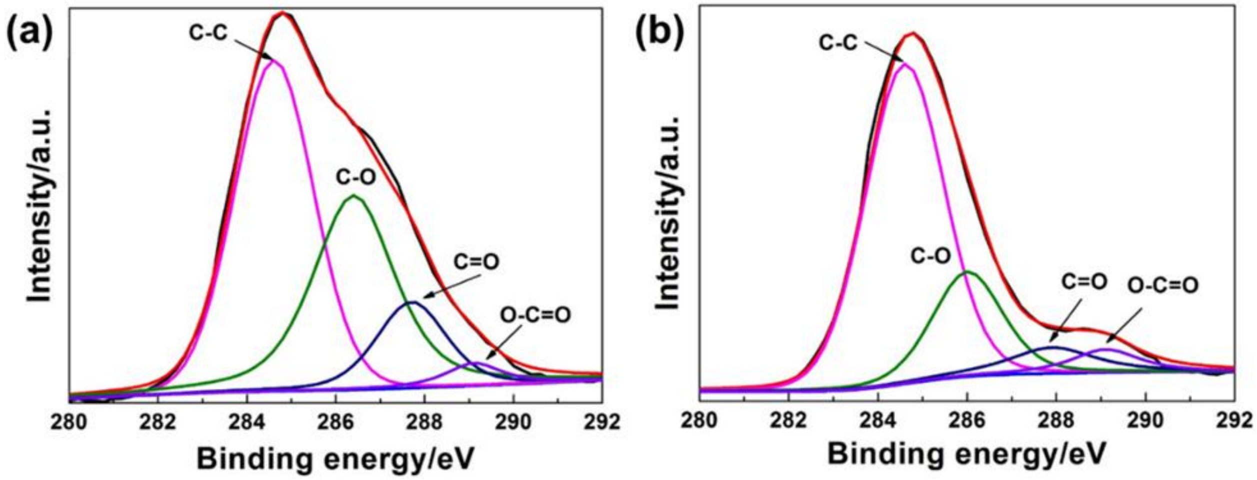

Figure S2. XPS patterns of GO-QDs (**a**) and GQDs (**b**).

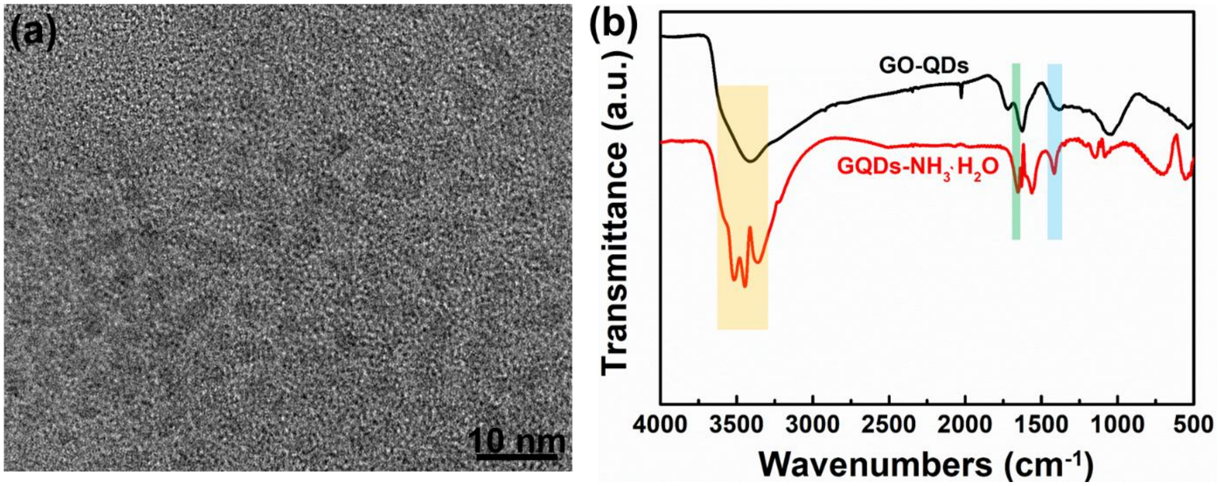

Figure S3. (**a**) TEM image of GQDs-$NH_3 \cdot H_2O$.and (**b**) FTIR spectra of GQDs-$NH_3 \cdot H_2O$ and GO-QDs.
